# Application of Ground-Based LiDAR for Analysing Oil Palm Canopy Properties on the Occurrence of Basal Stem Rot (BSR) Disease

**DOI:** 10.1038/s41598-020-62275-6

**Published:** 2020-04-15

**Authors:** Nur A. Husin, Siti Khairunniza-Bejo, Ahmad F. Abdullah, Muhamad S. M. Kassim, Desa Ahmad, Aiman N. N. Azmi

**Affiliations:** 1 Department of Biological and Agricultural Engineering, Faculty of Engineering, Universiti Putra Malaysia, 43400 UPM Serdang, Selangor Malaysia; 2 Smart Farming Technology Research Centre, Universiti Putra Malaysia, 43400 UPM Serdang, Selangor Malaysia

**Keywords:** Image processing, Environmental impact

## Abstract

Ground-based LiDAR also known as Terrestrial Laser Scanning (TLS) technology is an active remote sensing imaging method said to be one of the latest advances and innovations for plant phenotyping. Basal Stem Rot (BSR) is the most destructive disease of oil palm in Malaysia that is caused by white-rot fungus *Ganoderma boninense*, the symptoms of which include flattening and hanging-down of the canopy, shorter leaves, wilting green fronds and smaller crown size. Therefore, until now there is no critical investigation on the characterisation of canopy architecture related to this disease using TLS method was carried out. This study proposed a novel technique of BSR classification at the oil palm canopy analysis using the point clouds data taken from the TLS. A total of 40 samples of oil palm trees at the age of nine-years-old were selected and 10 trees for each health level were randomly taken from the same plot. The trees were categorised into four health levels - T0, T1, T2 and T3, which represents the healthy, mildly infected, moderately infected and severely infected, respectively. The TLS scanner was mounted at a height of 1 m and each palm was scanned at four scan positions around the tree to get a full 3D image. Five parameters were analysed: S200 (canopy strata at 200 cm from the top), S850 (canopy strata at 850 cm from the top), crown pixel (number of pixels inside the crown), frond angle (degree of angle between fronds) and frond number. The results taken from statistical analysis revealed that frond number was the best single parameter to detect BSR disease as early as T1. In classification models, a linear model with a combination of parameters, ABD – A (frond number), B (frond angle) and D (S200), delivered the highest average accuracy for classification of healthy-unhealthy trees with an accuracy of 86.67 per cent. It also can classify the four severity levels of infection with an accuracy of 80 per cent. This model performed better when compared to the severity classification using frond number. The novelty of this research is therefore on the development of new approach to detect and classify BSR using point clouds data of TLS.

## Introduction

LiDAR (Light detection and ranging) is an active remote sensing technology similar to Radar (Radio detection and ranging) but which uses laser light. LiDAR measures the distance or range to a target by illuminating the target with a pulsed laser light and measuring the reflected pulses with a sensor. It can directly represent external structures and carry out profiling for objects or trees. Laser Scanning (LS) profiling systems consist of a measuring instrument that can measure vertical angles, horizontal angles, and distances with a high standard of accuracy and speed by means of a mobile mirror or prism system allowing the mapping of morphological features of targets^1^.

Research and field site works used extensive biometric data in estimating tree properties while offering the possibility of reducing inventory costs. Previous studies have demonstrated that LiDAR could be used to derive canopy vegetation profiles and other structural trees’ properties from an understorey perspective. The point cloud resulted from LiDAR can yield information on a tree’s attributes such as height, canopy area, basal area, stem volume and fronds properties^2–4^. The systems can be deployed quickly in several locations and can gather information to measure unique attributes faster than those collected by field workers^5^. Balduzzi^6^ stated that research in remote sensing suggested that the micro differences visible in the point clouds analysis could be used to detect physical and external changes of the tree including the possibility of disease. It can be concluded that TLS well adapted for intensive study of the tree geometries. Irrespective of the type of LiDAR platform - terrestrial, mobile or airborne - this enabled the quantification of the 3D structure of tree canopies in a cost-effective, rapid and accurate manner^7,8^.

**Figure 1:**
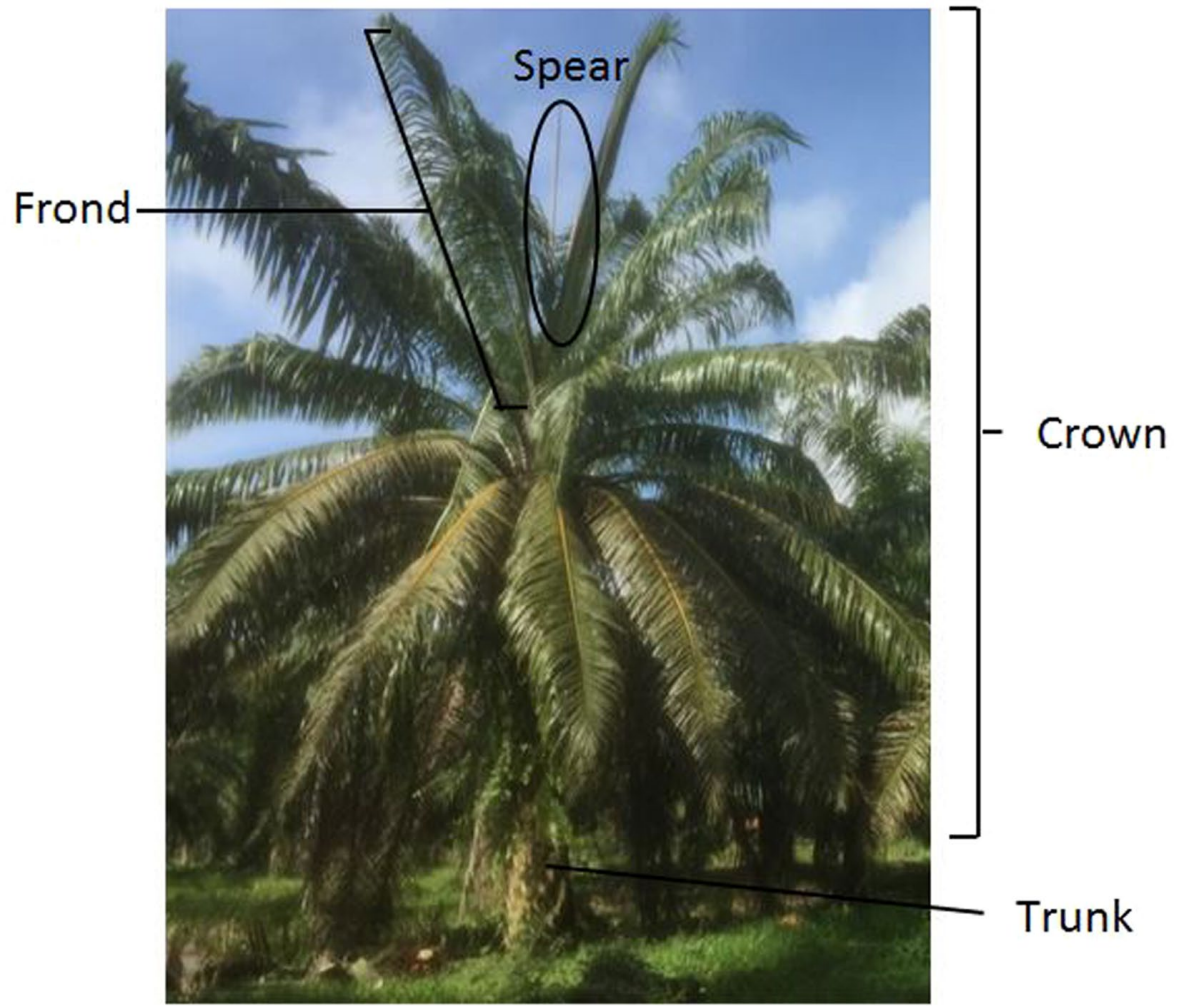
Generalized palm morphology.

The oil palm is a member of the monocotyledonous palm family (Arecaceae) (Fig. 1). It is a perennial tree crop, which better resembles a forest tree than other agricultural crops^9^ and, as an industrial crop, oil palms are planted in monoculture fashion. In most commercial plantations, the majority of planted oil palms are tissue culture clones, which makes the oil palms appear uniform except for abnormal palms^10^. The woody stem carries a single terminal growing point, from which leaves appear at regular intervals in a double spiral^11^. A multilayer canopy is made with old leaves that have progressively been covered by new leaves, subsequently forming the crown. Oil palm tree always develops one new leaf or frond at a time and on average one leaf is produced per month until the seedling is 6 months old. The leaf production increases to 30 to 40 leaves per year up to 6 years old and then declines to 18 to 25 leaves^12^.

BSR caused by *Ganoderma boninense* pathogens has caused serious damage in Malaysia for more than 50 years, whereas in the past few decades it is spreading rapidly. The disease progresses slowly but eventually all infected plants die. One of the main problems is the disease cannot be detected in its early stages, and when the disease symptoms do appear more than 50 per cent of internal tissues are already rotten^13^. Detecting BSR infection in its early stage allows palms to be treated, avoiding more extensive damage to the tree. Specific BSR disease symptoms include the canopy hanging downward (known as “skirting”), yellowing colour of the fronds, wilting green fronds and reducing frond production that causes the small size of the canopy, appearance of unopened young leaves (known as spears), fractured old fronds and fungal fruiting bodies on the oil palm trunk^14–17^. According to Balduzzi^6^ the damages caused by fungal diseases have a visual impact on the plants and the damaging factor changes a tree’s geometry.

Table 1 summarises available methods used for BSR detection. Generally, it can be grouped into three approaches: manual, non-remote (lab-based) and remote (non-invasive) techniques. The manual detection is considerably low in accuracy and labour intensive^18^. Conversely, non-remote or lab-based methods are costly, complex, labour intensive and are not designed for outdoor conditions^19^. Various remote sensing approaches have been introduced to detect BSR either from ground, aerial or satellite platforms. However, until now there is no critical investigation on the characteristics of canopy architecture related to this disease using TLS method was carried out.

**Table 1:** Summary of methods used for BSR detection.

Methods	Description	Features	References
Manual	Visual inspection	Canopy, trunk and root	^50^
Non-remote (Lab based)	Selective medium (GSM) Polyclonal antibodies (PAbs) Ethylenediaminetetraacetic acid (EDTA) Enzyme-linked immunosorbent assay (ELISA)-PAbs Polymerase chain reaction (PCR)	Trunk’s or root’s tissues	^44^ ^51,52^ ^53^ ^54^ ^55^
Remote (Non-invasive)	Electronic nose Tomography Microfocus x-ray Electrical properties Colour spectral Satellite image Thermal image Radar Hyperspectral Terrestrial laser scanning (TLS)	Trunk’s odour Trunk’s properties Leaf Leaf and trunk Leaf spectral Canopy spectral Canopy Canopy Leaf spectral Canopy spectral Canopy and trunk	^56,57^ ^58–61^ ^62^ ^63,64^ ^65^ ^32^ ^45^ ^31^ ^10,14,17,66–68^ ^33,34^ ^18^

Terrestrial Laser Scanner (TLS) uses laser energy to penetrate through canopy gaps and measure canopy structure^20^, while optical imagery cannot see the lower part of the tree’s canopy. The advantages of the TLS technique include easy and fast data acquisition (e.g. minutes), high accuracy, simple set up and high resolution^21^. TLS allows imaging from different perspectives and repeatable views, and offers only a one-time cost of equipment purchase. It is specially designed for outdoor application, is easily portable (relatively small size and low weight)^22^ and suitable to be used in an oil palm plantation. TLS sensors are considered a cheaper and affordable method of remote sensing and are easy to use by both non-professional and professional users^23^.

It is hypothesised that healthy trees have larger crown sizes and well-developed canopies compared to infected trees^24–26^. BSR infection could cause changes to the physical appearance and growth of oil palm trees. The changes are due to the damage to the internal tissue of trees, which restrict the water and nutrient consumptions, consequently disrupting tree growth and degenerates the physical condition of oil palm trees^27^. Infected trees also have less ability to perform normal photosynthesis compared to uninfected trees due to foliar symptoms and water deficiencies^28^. The disease at an advanced stage causes more dangled fronds and canopy hanging down than to a skirt structure^29^. Meanwhile, stunted leaf growth, delayed of new leaf development and reduced frond production leads to a smaller crown size^30^. The impact of the disease on the tree’s physical structure is more pronounced and detectable depending on the severity of the infection. Therefore, the differences in physical descriptions of oil palm trees such as level of canopy drooping, crown coverage area and frond features can be studied using TLS method. The study could be used to differentiate between healthy and infected trees, and to classify the trees in different stages of BSR infection towards an early detection approach.

The first study on the use of TLS for BSR detection was performed by Khairunniza-Bejo and Vong^18^. The results showed that there were correlations between the oil palm trunk’s perimeter, Diameter-Based Height (DBH) and canopy area with the BSR disease. This preliminary study supported the potential use of TLS for analysing the properties of oil palm trees to distinguish healthy and infected BSR at different levels of infection. Therefore, the objectives of this study are: 1) to analyse the canopy architectures based on the laser point density at different canopy levels, number of pixels inside the crown (crown pixel), degree of angles between tree fronds (frond angle) and number of fronds (frond number) of healthy and infected BSR, and 2) to develop classification models to detect severity levels of BSR using all physical properties extracted from TLS data. The novelty of this research is therefore on the development of a BSR detection model by using physical features extracted from TLS data which can classify BSR at 4 different levels of infection.

## Results and Discussion

### Basic information about the data

Table 2 shows the basic information for the scanned trees. In general, all trees have almost similar height and crown length ranging from 11.173 to 11.763 m and from 10.097 to 10.684 m, respectively. It is also shown that T0 has the lowest mean of crown length and the highest mean of crown width with 10.097 m (sd = 1.21 m) and 11.649 m (sd = 2.055 m), respectively, and T1 has the highest mean of height and lowest mean of crown width with 11.763 m (sd = 0.869 m) and 10.684 m (sd = 1.09 m), respectively. Furthermore, Table 3 shows the mean and variation of another 40 oil palm trees taken from Plot 4 that were used in validation of classification methods. Overall, all trees are of almost similar height and crown length, ranging from 11.893 m (sd = 1.056 m) to 11.023 m (sd = 1.346 m), and from 10.786 m (sd = 1.290 m) to 10.223 m (sd = 1.136 m), respectively.

**Table 2:** Mean and standard deviation of the data used to create the models.

Levels	Height (m)	Parameters Crown length (m)	Crown width (m)
T0	11.536 ± 1.084	10.097 ± 1.210	11.649 ± 2.055
T1	11.763 ± 0.869	10.684 ± 1.090	11.277 ± 1.426
T2	11.401 ± 0.880	10.660 ± 0.945	10.402 ± 1.598
T3	11.173 ± 1.235	10.243 ± 1.254	9.599 ± 0.850

**Table 3:** Mean and standard deviation of the data used to validate the models.

Levels	Height (m)	Parameters Crown length (m)	Crown width (m)
T0	11.893 ± 1.056	10.786 ± 1.290	11.899 ± 1.198
T1	11.456 ± 0.796	10.552 ± 1.119	11.326 ± 1.349
T2	11.320 ± 0.775	10.253 ± 1.045	9.899 ± 1.446
T3	11.023 ± 1.346	10.223 ± 1.136	9.463 ± 1.124

### Canopy description

**Figure 2:**
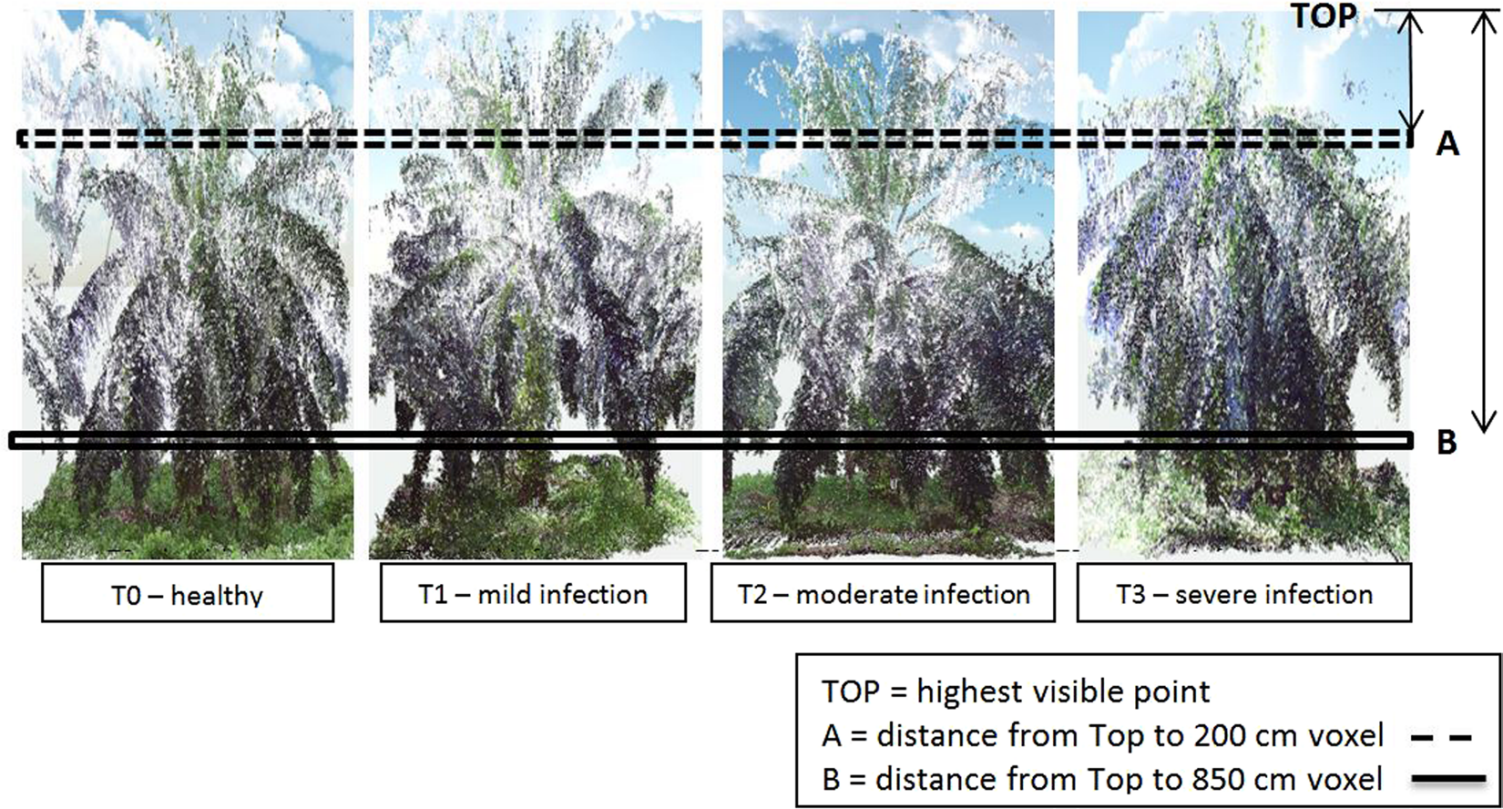
Coloured point clouds show the positions of S200 and S850.

Canopy stratification analysis was performed to identify the canopy level, which gave a significant difference between healthy and infected trees. One of the most prominent and distinctive BSR symptoms on the canopy is the “skirting” effect. Thus, the difference of canopy density at specific levels could be used to study the drooping or flattening effects of the canopy caused by *Ganoderma* infection. 3D images of oil palm trees were constructed using the laser point data and the canopy was stratified horizontally. Data gathered from 40 oil palm trees showed some variations of laser point density from the top of the canopy to the bottom. The top is defined by the highest visible point of a tree and the bottom is defined by the lowest visible point of a tree. Strata with very few leaves visible at the bottom part of the box provided very low voxel (volume element) values or extreme values, which biased the statistical analysis. In addition, any layers located below 9 m for its entirely was excluded because it likely represented ground level vegetation. Considering this factor, only strata from 0 cm to 850 cm from the top were analysed. Results taken from the Kruskal-Wallis test (Table 4) revealed that only strata at 200 cm from the top (known as S200) and strata at 850 cm from the top (known as S850) (Fig. 2) were significant for all levels of health (T0 to T3) at the 5 per cent significance level.

**Table 4:** Analysis of variance (Kruskal-Wallis) for every parameter.

Parameters	Chi-square value	p-value
S200	10.248	0.0166*
S850	8.058	0.0471*
Crown pixel	23.058	<0.0001*
Frond Angle	32.666	<0.0001*
Frond Number	33.428	<0.0001*

*significant at 5% level.

### Statistical analysis

The one-way analysis of variance results using Kruskal-Wallis test found that all parameters varied significantly between each level with a p-value less than 0.05 (Table 4). Crown pixel, frond angle and frond number yielded a p-value, <0.0001, while S200 and S850 yielded a p-value of 0.017 and 0.047, respectively. The highest chi-square value was obtained by frond number (33.428) and the lowest was obtained by S850 (8.058).

### Classification severity

The six types of classification models developed in this study were linear, two-factorial, quadratic, cubic, quartic and fifth models. Linear model used single parameters in the equation model. Two-factorial model used the interaction term of two parameters. Meanwhile, quadratic, cubic, quartic and fifth models pertains to the second, third, fourth and fifth order with an equation using polynomial function of degree 2, 3, 4 and 5, respectively. Twenty six combinations of parameters were developed for every linear, two-factorial and quadratic models, 20 models were developed for cubic and 10 combinations were developed for quartic and fifth models using the Design Expert software. Less number of combinations was developed for higher degree of polynomial function i.e. cubic, quartic and fifth models because the models were too complex. Coded for each parameters and coded combination of parameters are tabulated in Table 5. In total, 118 classification models were developed using all the parameters.

**Table 5:** Coded for each parameter and coded combination of parameters.

Coded for parameters	Coded combination of parameters			
	Two	Three	Four	Five
A = Frond number	AB, AC,	ABC, ABD,	ABCD,	ABCDE
B = Frond angle	AD, AE,	ABE, ACD,	BCDE,	
C = Crown pixel	BC, BD,	ACE, ADE,	ACDE,	
D = S200	BE, CD, CE,	BCD, BCE,	ABDE,	
E = S850	CD	BDE, CDE	ABCE	

The values obtained from each equation were used to find differences between each healthiness level by deduction of T1 with T0, T2 with T1 and T3 with T2. The best model is defined as the model that could provide the largest differences between the levels and with the smallest or none overlapping in the range values, especially between T0 and T1. The latter is important to separate between the healthy and unhealthy levels. Moreover, the larger the difference shows better separation between the levels. Table 6 shows an example of best combination selection using linear model. The best combination of parameters for linear model is ABD, which consists of frond number, frond angle and S200 parameters. This combination has the largest dfference between T1 and T0, which is equal to 1.865.

**Table 6:** Equation’s value and difference between healthiness level for linear model.

Combined properties	Values and difference between healthiness level						
	T0	(T1 - T0)	T1	(T2 - T1)	T2	(T3 - T2)	T3
AB	−0.787	1.826	1.039	1.397	2.436	0.266	2.703
AC	−0.472	1.403	0.931	1.408	2.339	0.205	2.544
AD	−0.667	1.657	0.989	1.442	2.431	0.279	2.710
AE	−0.541	1.640	1.099	1.461	2.560	0.277	2.836
BC	0.382	0.388	0.771	1.245	2.016	0.136	2.152
BD	0.297	0.591	0.888	1.136	2.024	0.279	2.303
BE	0.547	0.705	1.252	1.493	2.745	0.297	3.041
CD	0.679	0.077	0.756	1.356	2.112	−0.119	1.993
CE	0.971	0.122	1.093	1.452	2.545	−0.099	2.446
DE	1.562	0.521	2.083	1.353	3.436	0.119	3.555
ABC	−0.589	1.546	0.957	1.429	2.386	0.206	2.592
**ABD**	−**0.843**	**1.865**	**1.023**	**1.486**	**2.508**	**0.273**	**2.781**
ABE	−0.693	1.781	1.088	1.451	2.539	0.271	2.810
ACE	−0.412	1.419	1.007	1.486	2.492	0.212	2.704
ACD	−0.512	1.419	0.907	1.503	2.410	0.209	2.620
ADE	−0.588	1.664	1.077	1.535	2.612	0.283	2.894
BCD	0.315	0.433	0.748	1.357	2.105	0.141	2.246
BCE	0.518	0.526	1.043	1.533	2.576	0.177	2.753
BDE	0.498	0.734	1.232	1.557	2.789	0.302	3.091
CDE	0.821	0.207	1.027	1.624	2.652	−0.079	2.573
ABCD	−0.639	1.573	0.934	1.528	2.463	0.210	2.673
ABCE	−0.501	1.504	1.004	1.479	2.483	0.210	2.694
ABDE	−0.764	1.827	1.063	1.527	2.591	0.276	2.867
ACDE	−0.458	1.433	0.976	1.571	2.547	0.215	2.762
BCDE	0.454	0.558	1.012	1.617	2.629	0.179	2.808
ABCDE	−0.569	1.541	0.971	1.566	2.537	0.214	2.750

Furthermore, the best combinations of two-factorial was BDE (frond angle-S200-S850), for quadratic was ABCD (frond number-frond angle-crown pixel-S200), for cubic was ACD (frond number-frond angle-crown pixel), for quartic was AC (frond number-crown pixel) and for fifth model was CE (crown pixel-S850). The mean value, standard deviation and the maximum and minimum that were used to set the range for upper and lower limits for every polynomial model is shown in Table 7.

**Table 7:** Descriptive statistics for all models.

Model	Level	Mean	Standard deviation	Mean + sd, Mean - sd	Max, Min	Range (Max-Min)	Range of classification
Linear	T0	−0.879	0.702	−0.176, 0.702	−0.138, −2.289	2.150	≤−0.176
	T1	1.272	0.284	1.556, 0.284	1.761, 0.939	0.821	0.177–1.556
	T2	1.900	0.479	2.379, 0.479	2.572, 1.205	1.368	1.557–2.379
	T3	2.515	0.390	2.904, 0.390	3.089, 2.144	0.945	≥2.38
Two factorial	T0	0.466	0.371	0.836, 0.095	1.417, 0.032	1.386	≤ 0.095
	T1	1.088	0.319	1.408, 0.769	1.635, 0.567	1.068	0.096–1.408
	T2	1.708	0.714	2.421, 0.994	3.269 1.027	2.241	1.409–3.141
	T3	2.174	0.968	3.142, 1.207	4.315, 1.254	3.061	≥3.142
Quadratic	T0	−7.590	3.944	−3.646, −11.533	−1.593, −12.412	10.819	≤−3.646
	T1	−1.056	2.882	1.826, −3.939	1.190, −7.024	8.214	−3.647–1.826
	T2	0.416	1.712	2.129, −1.296	2.314, −4.055	6.369	1.827–2.418
	T3	1.167	1.252	2.419, −0.085	2.891, −1.648	4.539	≥2.419
Cubic	T0	−6.857	2.900	−3.956, −9.757	−1.040, −9.852	8.812	≤−3.956
	T1	0.619	3.536	4.156, −2.917	6.692, −7.450	14.142	−3.957–1.952
	T2	2.174	2.682	4.856, −0.508	9.562, 0.054	9.508	1.951–2.897
	T3	2.139	0.759	2.898, 1.380	3.457, 1.111	2.347	≥2.898
Quartic	T0	4.274	3.020	7.294, 1.255	6.900, −2.858	9.758	≥4.274
	T1	2.643	1.857	4.500, 0.786	5.700, 0.856	4.844	4.275–2.100
	T2	1.975	1.574	3.549, 0.401	5.700, 3.549	4.991	≤2.727
	T3	2.727	1.436	4.163, 1.291	6.200, 4.163	4.833	2.101–2.727
Fifth	T0	4.859	3.476	8.335, 1.383	9.782, 0.331	9.451	≥4.859
	T1	1.608	3.144	4.752, −1.536	8.032, −2.617	10.649	4.860–2.614
	T2	0.421	2.580	3.000, −2.159	3.313, −5.461	8.774	≤0.421
	T3	2.615	5.711	8.326, −3.097	11.095, −4.374	15.469	2.615–0.420

Table 8 shows lists of correctly classified trees, misclassified trees, accuracy and average accuracy for the best model in linear, two-factorial, quadratic, cubic, quartic and fifth models. Linear, quadratic and cubic models have the highest accuracies for the healthy tree classification (100% accuracy). Although quadratic and cubic models can classify healthy oil palm trees with an accuracy of 100%, the average accuracies for all severity levels are lower compared to the linear model (60% and 62.5% compared to 80%). The misclassifications only occurred among the unhealthy groups where T1 misclassified as T2 and T3, T2 was misclassiied as T1 and T3, and T3 was misclassified as T1 and T2. Linear model presented the highest accuracy for T1 classification and two-factorials presented the highest accuracy for T3 classification (100% accuracy). In fifth model, the classification was mixed up between the healthy and unhealthy levels. In addition, the average accuracy was the lowest with 30% for all levels and 36.67% for healthy-unhealthy classification. It was possible the model for fifth order was too complex and it could not accurately classify the oil palm trees. Classification of T2 level could not achieve 100% accuracy in all models; the highest classification was 70% in two-factorial model. It could be due to the rank of T2 at the middle of unhealthy class. Thus, the transition from mild infection to moderate infection and the transition from moderate infection to severe infection might be difficult to separate. It is also possible that the ranges between the levels were very close, which could cause the misclassification of oil palm trees. However, the classification model can still differentiate between the healthy and the infected trees. Frond number was used to create a simple severity classification using a single parameter because it was the best parameter among the five parameters analysed. The classification using frond number for healthy trees achieved 100% accuracy, while the average accuracy for unhealthy trees was 63.33%. The average accuracy for severity classification was 72.5% and the average accuracy for healthy-unhealthy classification was 81.67%.

**Table 8:** Accuracy of the classification models.

Type of model	Parameters	Level	Healthiness Level					
			T0	T1		T2		T3
Linear	ABD	Correctly classified tree (%)	100	100		60		60
		Misclassified as	—	—		4 T1		4 T2
		Average accuracy (%)	80					
		Healthiness level	Healthy	Unhealthy				
		Correctly classified tree (%)	100	73.33				
		Average accuracy (%)	86.67					
		Level	T0	T1	T2			T3
Two factorial	BDE	Correctly classified tree (%)	90	70	70			100
		Misclassified as	1 T2	1 T0, 1 T2, 1 T3	2 T1, 1 T3			—
		Average accuracy (%)	82.5					
		Healthiness level	Healthy	Unhealthy				
		Correctly classified tree (%)	90	80				
		Average accuracy (%)	85					
Quadratic	ABCD	Level	T0	T1	T2		T3	
		Correctly classified tree (%)	100	40	30		70	
		Misclassified as	—	3 T2, 3 T3	4 T1, 3 T3		1 T1, 2 T2	
		Average accuracy (%)	60					
		Healthiness level	Healthy	Unhealthy				
		Correctly classified tree (%)	100	46.67				
		Average accuracy (%)	73.33					
Cubic	ACD	Level	T0	T1		T2		T3
		Correctly classified tree (%)	100	50		50		50
		Misclassified as	—	5 T2		3 T1, 2 T3		2 T1, 3 T2
		Average accuracy (%)	62.50					
		Healthiness level	Healthy	Unhealthy				
		Correctly classified tree (%)	100	50				
		Average accuracy (%)	75					
Quartic	AC	Level	T0	T1	T2			T3
		Correctly classified tree (%)	90	50	40			70
		Misclassified as	1 T3	4 T2, 1 T3	4 T1, 2 T3			3 T2
		Average accuracy (%)	62.50					
		Healthiness level	Healthy	Unhealthy				
		Correctly classified tree (%)	90	53.33				
		Average accuracy (%)	71.67					
Fifth	CE	Level	T0	T1	T2		T3	
		Correctly classified tree (%)	50	0	10		60	
		Misclassified as	5 T3	9 T0, 1 T3	8 T0, 1 T3		4 T0	
		Average accuracy (%)	30					
		Healthiness level	Healthy	Unhealthy				
		Correctly classified tree (%)	50	23.33				
		Average accuracy (%)	36.67					
Single	A	Level	T0	T1	T2		T3	
		Correctly classified tree (%)	100	90	50		50	
		Misclassified as	—	1 T0	5 T1		5 T2	
		Average accuracy (%)	72.5					
		Healthiness level	Healthy	Unhealthy				
		Correctly classified tree (%)	100	63.33				
		Average accuracy (%)	81.67					

Linear model was chosen as the best parameters because it could detect 100% of healthy trees in severity-level classification and in healthy-unhealthy classification, and it also delivered high average accuracies for both classifications, 80% and 86.67%, respectively. Although quadratic and cubic models also achieved 100% accuracy for the healthy tree classification, the average accuracies for both classifications were lower than the linear model. Similar to two-factorial model, even though the average accuracy for the severity level classification was higher than the linear model (82.5%), the model did not achieve 100% accuracy in healthy tree classification and had lower average accuracy in healthy-unhealthy classification compared to linear model. Thus, the results of linear model were more consistent. Compared to the best single parameter, even though frond number and linear model produced 100% accuracy in healthy tree classification, the average accuracy in four severity level classification for linear model was 80%, higher than frond number of 72.5%. Linear model also performed better than frond number in healthy-unhealthy classification with 86.67% accuracy compared to 81.67%. Compared to other polynomial model, linear model has unbiased fit and the convergence rates of linear are the most efficient. For non-linear models, the fitted relationship is difficult and the regression is usually under or over predicted and sometimes the constraints in non-linear models can fail to converge. The results show that the linear model was the best model to be used for severity detection and classification encompasses of ABD i.e., by using frond number (A), frond angle (B) and S200 (D) parameters as shown in Eq. (1).

1 $${\rm{H}}{\rm{e}}{\rm{a}}{\rm{l}}{\rm{t}}{\rm{h}}{\rm{i}}{\rm{n}}{\rm{e}}{\rm{s}}{\rm{s}}\,{\rm{l}}{\rm{e}}{\rm{v}}{\rm{e}}{\rm{l}}=5.913-0{.243}^{\ast }{\rm{A}}-0{.0204}^{\ast }{\rm{B}}+4.424{\rm{e}}-{06}^{\ast }{\rm{D}}$$

where A = Frond number

B = Frond angle

D = S200

As shown in Table 9, the research work presented in this paper is the first approach of BSR detection using combination of canopy strata, crown area, frond number and frond angle extracted from TLS data. In canopy structure properties, the currently available methods used in the oil palm fields were focusing on canopy spectral taken from satellites images, airborne images and spectroradiometer. Some of the work also used leaf spectral taken from spectroradiometer. Meanwhile, other tree structures that were also considered include trunk using odour and tomography sensors. In overall, the available method could give accuracy between 77% to 100% when using canopy structure. It is shown that the proposed method with the percentage accuracy of 80% in 4 levels of healthiness and percentage accuracy of 86.67% in 2 levels of healthiness is acceptable as compared to currently available methods. It gave higher percentage of accuracies compared to canopy spectral approach using satellite images in 4 levels of healthiness^31^ and 2 levels of healthiness^32^ and higher percentage of accuracy compared to airborne images in 2 levels of healthiness^33,34^. This might due to the difference types of platform used during data collection. Data collected from the ground have less effect from the environmental factors i.e. weather conditions, sun illumination and cloud coverage. This study also showed a similar pattern with previous methods, where higher accuracy was obtained in two levels of infections compared to four levels of infections. It shows that it is easier to differentiate between healthy and unhealthy oil palm trees and it was more challenging to classify the disease into more levels of infections.

**Table 9:** Summary of the approach methods to detect BSR disease.

Oil palm part	Features	Level	Highest accuracy
Canopy	Canopy spectral (satellite)	4	77%^31^
		2	84%^32^
	Canopy spectral (airborne)	2	84%^33^
		2	86%^34^
	Canopy spectral (spectroradiometer)	4	94%^14^
		2	98%^14^
	Leaf spectral (spectroradiometer)	2	100%^66^
		4	97%^17^
	Canopy strata, crown area, frond number and frond angle (TLS data)	4	80% (Proposed method)
		2	86.67% (Proposed method)
Trunk	Odour	2	100%^56^
		2	100%^57^
	Tomography	2	100%^59^
		5	82%^60^

### Future work - large area implementation

This study’s findings highlight the potential of TLS for high-resolution 3D measurements of oil palm trees. It provides a very high level of detail, accurate, non-destructive, and have potential to be automated. It is the first comprehensive study of canopy characteristics using TLS for BSR disease. *In-situ* observation is laborious, subjective and depends on individual workers who may be prone to fatigue. TLS is considered as a simple and user-friendly technology for gathering information. The evolution of TLS technology will continue to enhance data quality, allowing for wider spatial coverage and faster scanning. On-site facilities could be established to provide an up-to-date database of the health of oil palm plantations.

The current approach was scanning a single tree at one time. Therefore, this method can be improved by scanning a group of trees at one time, where the time for scanning and processing the data can be reduced. The use of mobile laser scanning (MLS) can be considered to accelerate the process of data acquisition and for the large area implementation. The scan could run concurrently by attaching the mobile scanner to a tractor or trailer that is used for operation such as collecting the Fresh Fruit Bunch (FFB), ferlitization and etc. in the plantation. It is therefore, the process of plant scouting can be done more rapid and effective since it does not depending on the specific task of the worker. The data collection also could be done by attaching the scanner to swan robot for the time dependence scouting. It was shown by Bienert *et al*.^35^ that the MLS is able to provide similar data compared to TLS with reducing point density due to the kinematic recording. If an MLS mounted on a terrain vehicle was considered, a clear path with clear height in the plantation should be planned to avoid the collision with the fronds. However, the differences in static and mobile laser scanning system such as viewing angles, spatial resolutions, scanning area and distances with a high standard of accuracy and speed using an MLS system need to be taken into considerations.

For a large area implementation, the possible arrangement of laser scanner position in the oil palm plantation is shown in Supplementary Fig. S1 online. A full scan of tree requires 26 mins of scanning process including movement from tree to tree and the equipment setup. Thus, for a plantation that consists of 160 palms/ha, it takes about 3 days to complete the process. Meanwhile, the manual tree scouting requires 10 mins per tree, resulting around less than 2 days to complete a hectare. Instead of inspecting each of the tree in the whole plantation area, the current manual scouting was done by a random sampling in every 6 months depends on the potential risks in the area^36^. The plantation was divided into 10 plots for each hectare, which consists of 16 trees per plot for a plantation with average planting density of 160 palms/ha. Only one oil palm tree will be sampled for each plot. The sampling approach was done due to limited number of expert workers in this field. Furthermore, the workers need extensive time for training to become an expert. *In-situ* observations are subjective and depend on workers’ abilities. Some of the *Ganoderma boninense* symptoms are not visible causing a faulty detection especially when inexperienced workers did the inspection. Naked eyes observation involves continuous monitoring from one tree to the other that is both laborious and prone to fatigue. The issue on workers shortage in plantation sector, adding another problem. The new finding in this research not only can fill the research gap in laser scanning study but also can be the eye-opener to community in this area. Even though the current proposed approach is time consuming, however it has a bright future by considering evolution of laser scanning technology that continuously enhance its data quality, spatial coverage, scanning time and providing different type of platform.

Furthermore, the method can be extended by using different type of platform such as an Airborne Laser Scanning (ALS). Three parameters extracted from the top-view i.e. frond number, frond angle and crown pixel could be used as the basis for the application. The current semi-automatic counting of frond number and angle value has potential to be automatic using deep learning technique. The studies by Bazezew^37^ and Hopkinson *et al*.^38^ shown that both ALS and TLS data can characterise the tree canopy but ALS unable to characterise the understorey vegetation structure in detail. Thus, it could be beneficial to integrate both methods to produce more accurate data. Point clouds fusion and assimilation by ALS and TLS could be done by using same point references^39,40^ or assigning multiple TLS point clouds to ALS point clouds^41^. Detail study need to be done since the ALS and TLS are varied in terms of scanning mechanism, data capture mode, typical project size and obtainable accuracy and resolution.

## Materials and Methods

### Data collection

The field measurements were performed at an oil palm plantation in Seberang Perak, Malaysia. A total of 40 oil palm trees were selected with 10 trees for each health level randomly chosen from different locations from the same plot. The age of the oil palm trees was 9 years old while the height of the trees was between 10 to 11 meters, where the production was at its peak. The oil palm cultivar in this plantation is from Dura and Psifera (D x P), and the soil type is coastal. Measurements were taken in a small block in order to avoid variations of the environment and when the sky was clear and there was no rain and no wind. The method was later verified at another different oil palm plantation block.

The average rainfall rate in the area was 200.5 ± 121.7 mm/month with an average of 15.9 ± 3.5 rain-days/month in the year of 2017. Major rainfalls occurred in January and November, while July and August are the driest months. The monthly mean relative humidity was approximately 84.1 ± 2.6% and average temperature was 28.0 ± 0.56 °C with minimum value of 27.0 °C in November and maximum values of 28.7 °C in June and July. The maintenance of the oil palm planting, including fertilisation regime, fruit harvesting, pruning, and weed management are following the commercial oil palm estates practices. Adequate pruning of mature palms is to remove the dead or senescing leaves and to facilitate in accessing the fresh fruit bunch (FFB) due to a correct harvesting time. In commercial plantation, the pruning was done twice per year, where more pruning was done for older trees^42,43^. The planting density for the plot was 160 palms/ha and the palm are planted in an equilateral triangle pattern with a distance of 9 m x 9 m x 9 m. The effect of drought on the canopy drooping and the effect of etiolation on the frond angle were not discovered in this study. Other randomized factors such as other varieties of oil palm trees, yields for individual tree, different rates of fertilizers and others were also not considered in this study. Since there’s no variations on the samples, therefore the unhealthy condition of the oil palm trees were assumed due to the *Ganoderma boninense* infection and confirmed by *Ganoderma* Selective Medium (GSM)^44^ analysis.

**Figure 3:**
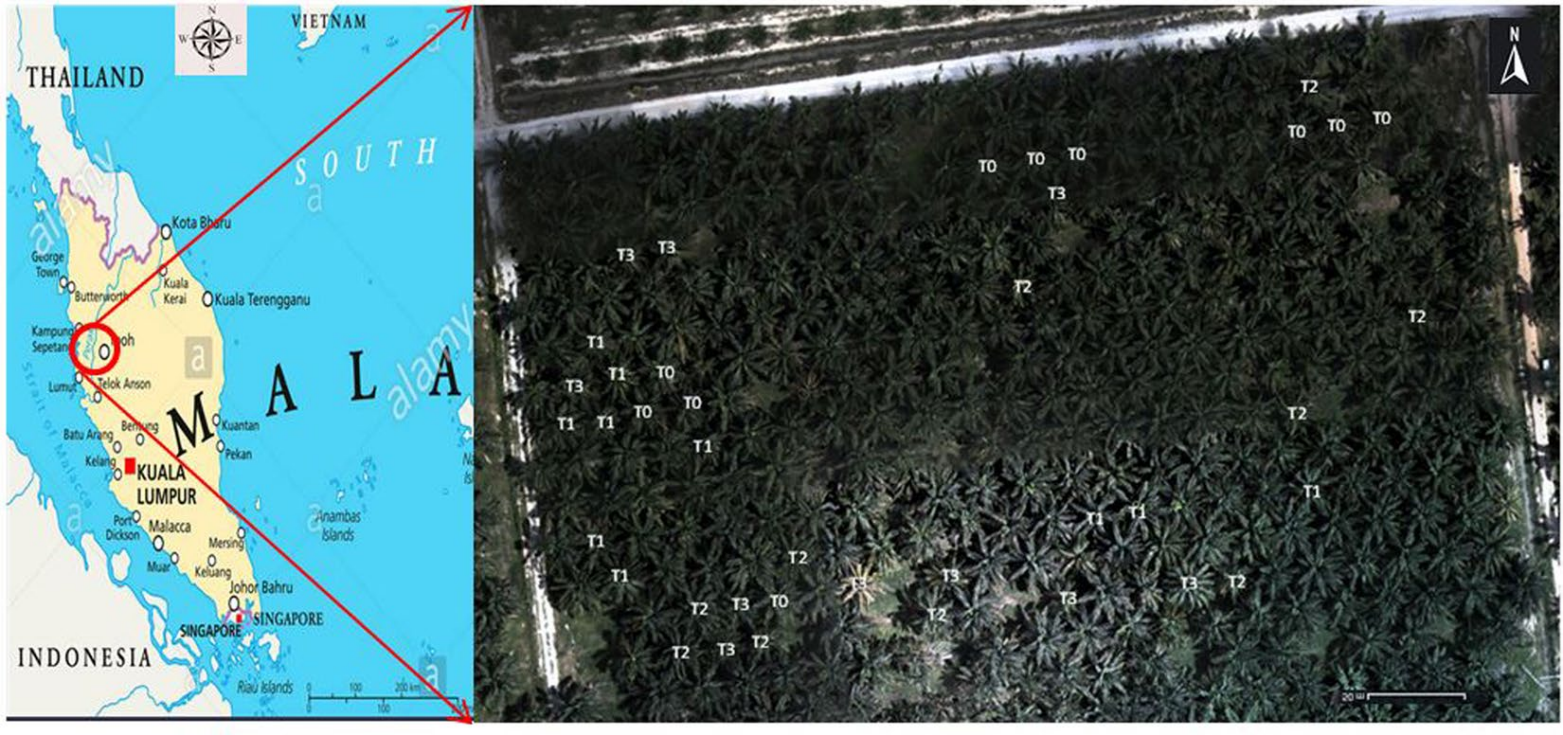
Location of study area and aerial map of tree’s location (map created by ENVI 5.1).

Data collection was conducted in early July 2017. The oil palm trees were categorised into four levels of health: T0 (healthy palm, no foliage symptom (0%), no fruiting body)), T1 (mild infection, no foliage symptom (0–25%), produce fruiting body), T2 (moderate infection, produce foliage symptom (25–50%), fruiting body) and T3 (severe infection, produce foliage symptoms (50–75%) and fruiting body)^45,46^. The subsequent results and discussions were based on these levels. The severity level was identified based on visual symptoms by experts from MPOB and were confirmed by GSM analysis. Figure 3 shows the aerial map of the trees’ location taken by 3DR Iris + drone with MAPIR Survey 2 camera (MAPIR, Peau Prodiuctions, Inc. CA, USA), where the trees were labelled based on their health levels.

**Figure 4:**
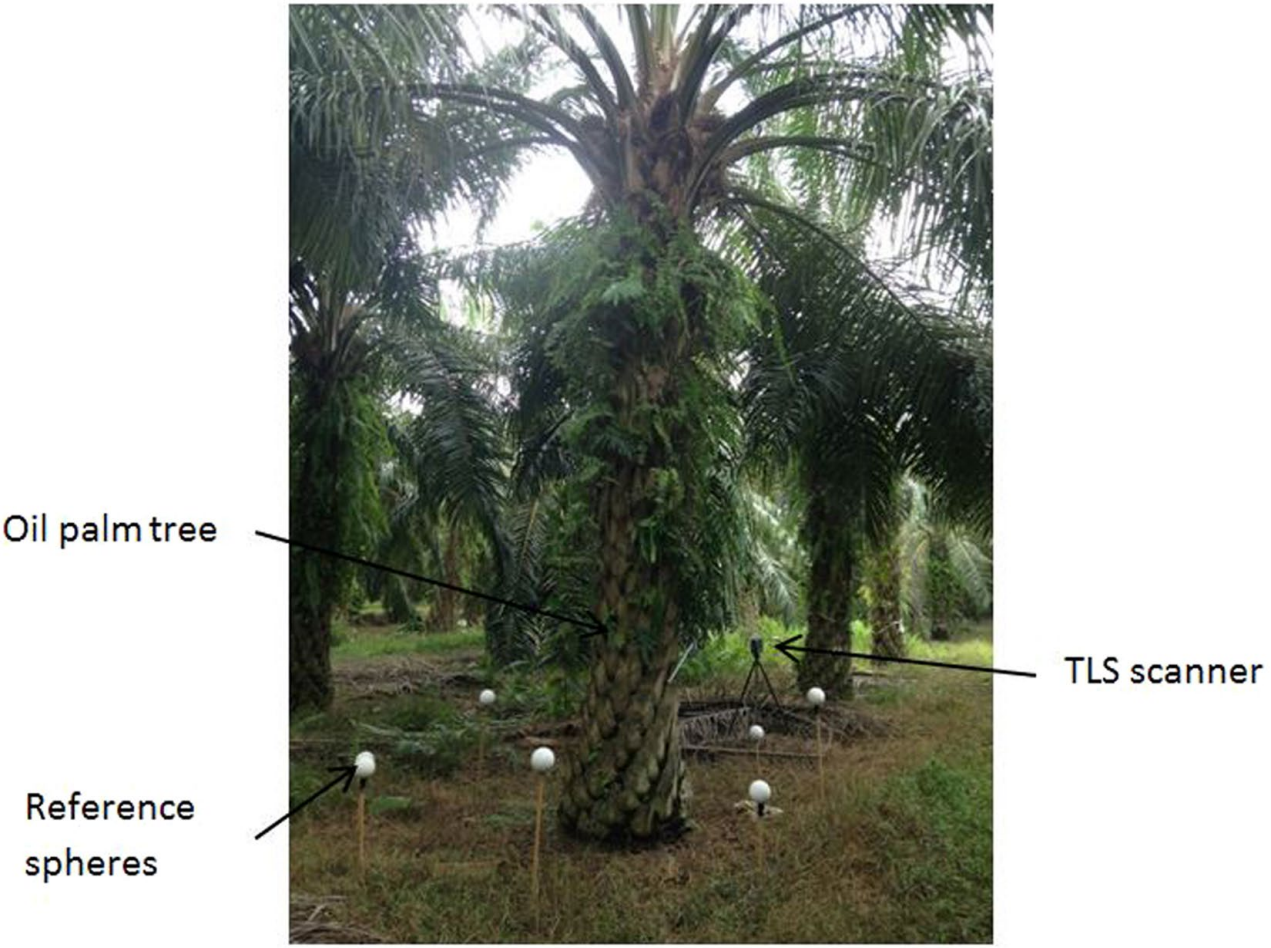
Setup of data collection.

Measurements of the tree were performed using a FARO Focus terrestrial laser scanner (Faro Technologies Inc., Florida, USA). The device used a laser beam and the “phase shift measurement technology” to detect distances. The distance of an object to the scanner was measured by analysing the shift in the phase of the returning beam. The laser rangefinder was mounted in the horizontal plane and aligned with the vertical axis of the instrument. The rotation of a mirror placed at 45° to the laser beam aperture (horizontal rotation) and the rotation of its trunnion (vertical rotation) provided a panoramic view of the scene - 305° × 360° - that surrounded the TLS as point cloud on a Cartesian or spherical basis. Therefore, by using ground scanning, the scanner had the capability to get a top-view of a tree. The TLS scanner was mounted on a surveying tripod at an estimated height of 1 m and a distance of 1.5 meters from a tree. The tripod was placed on firm ground and levelled using a bubble balancer. Each palm was scanned at four different positions around the tree (Fig. 4). The laser scan was recorded on a removable SD memory card and then transferred to the SCENE software (version 6.2, FARO Technologies, Inc.) for processing. SCENE is FARO’s point clouds manipulation software specifically designed to process and manage the scan data. Details on the features of the TLS FARO Focus are shown in Table 10.

**Table 10:** The features provided by the TLS FARO Focus.

Measurement principle	Specifications
Field of View (vertical × horizontal)	305° × 360°
Wavelength	905 nm (Infrared light spectrum)
Diameter beam aperture	3 mm, circular
Beam divergence	0.015°
Sensor FOV	0.27 mrad
Range	0.6 m–120 m
Ranging error (Accuracy)	±2 mm

### Point cloud registration

**Figure 5:**
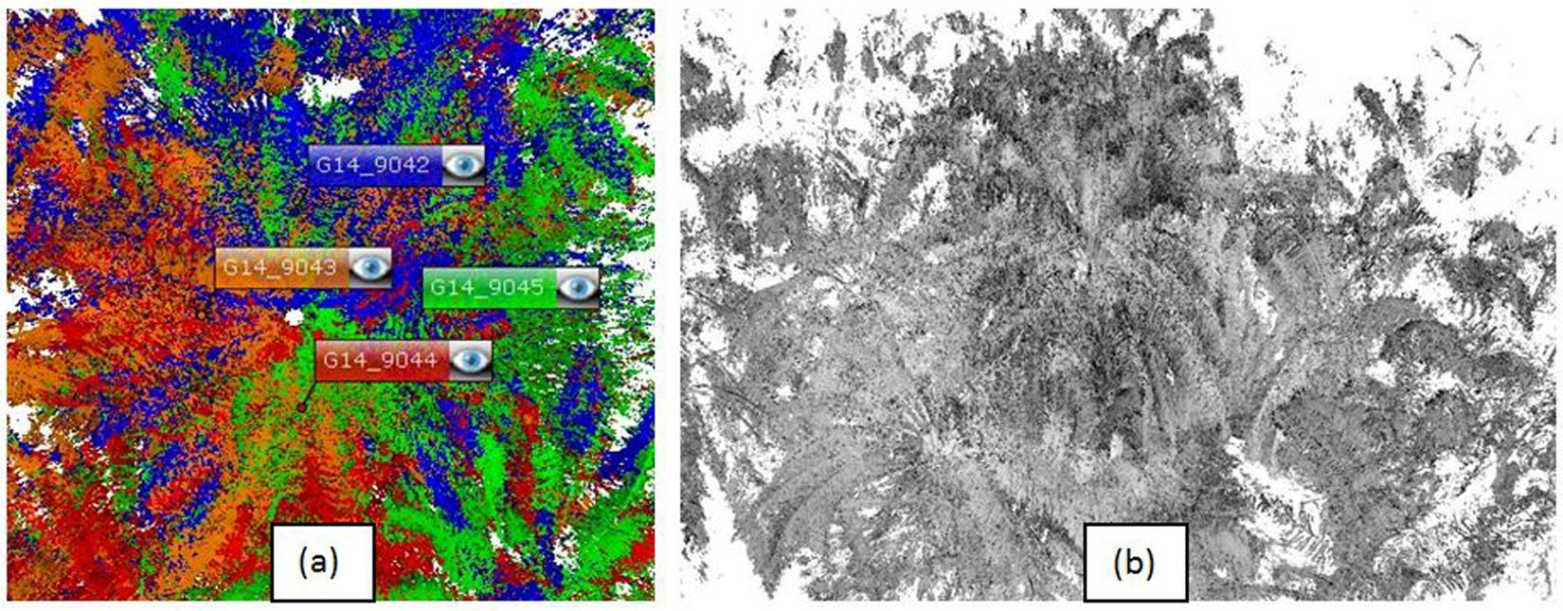
(**a**) Cluster of point clouds before registration, (**b**) 3D view of point clouds after registration.

All collected scans were imported into the SCENE software for processing and part of the analysis. ‘Registration’ tab was used for registration and automatic registration was chosen from the three options available, including manual and visual. Automatic registration offers more convenient and better accuracy when used together with ‘top view and cloud-to-cloud’ methods. Based on the recorded scan position and its location, confirmation of the clustered point clouds could then be verified. Multiple TLS scans were matched and the laser point data were synced to create a cluster of point clouds and a complete 3D view of the tree. After that, the cluster of scan points could be viewed in ‘3D view’ tab (Fig. 5).

### Single tree extraction

**Figure 6:**
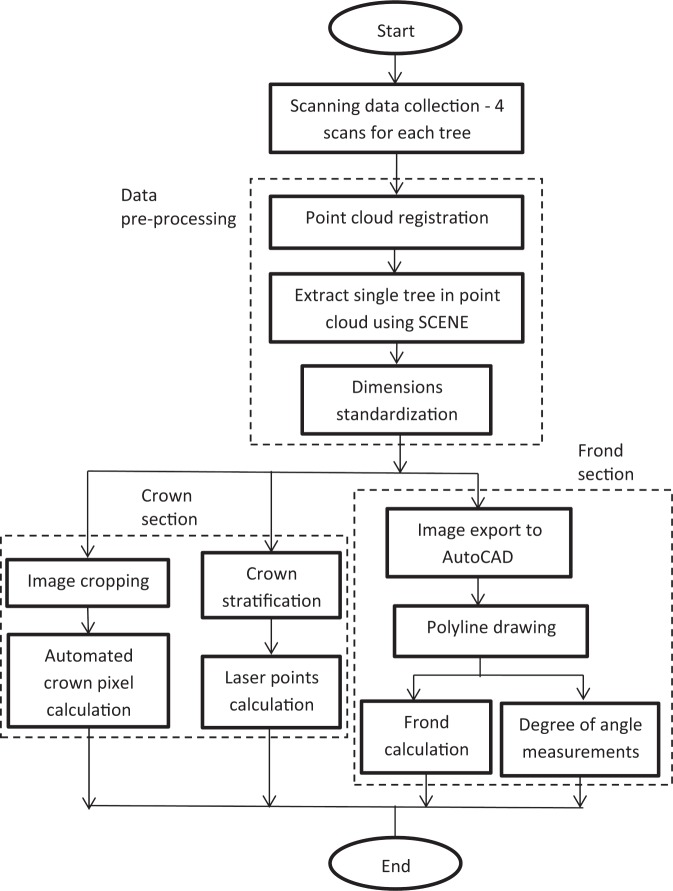
Flowchart of the methods.

Figure 6 shows the flowchart of the step-by-step method used in the study for the crown, frond and canopy. A clipping box was created to isolate the tree from the background. In order to get a top view of the whole image of an oil palm tree, the cursor of the clipping box was moved upwards to the uppermost part of the canopy tree and was then moved down to the bottom limit (before the ground). Thus, the whole tree sample could be seen from different views - left, right, front, back and isometric using the box.

### Crown section

The length and width of the box from top view were set to 10 m for size standardisation. The clipping box was viewed in ‘top view’ before being saved in JPEG format. Then, the image was cropped using Paint software (Microsoft, Washington, USA) to remove unwanted features such as fronds from other trees and was later processed in Matlab (Mathworks Inc., Massachusetts, USA) software. Region-based segmentation using the Otsu’s algorithm was used to separate the crown from the background. The process involved portioning the crown area by subdividing the image into similar areas of connected pixels. Otsu is a type of global thresholding which relies on the grey value of the image to separate objects of interest in an image from the background based on their grey-level distribution. The algorithm assumes that the image contains two classes of pixels following a bi-modal histogram (foreground pixels and background pixels). It then calculates the optimum threshold separating the two classes so their combined spread (intra-class variance) is minimal. The method is widely used because it is simple and effective^47^.

**Figure 7:**
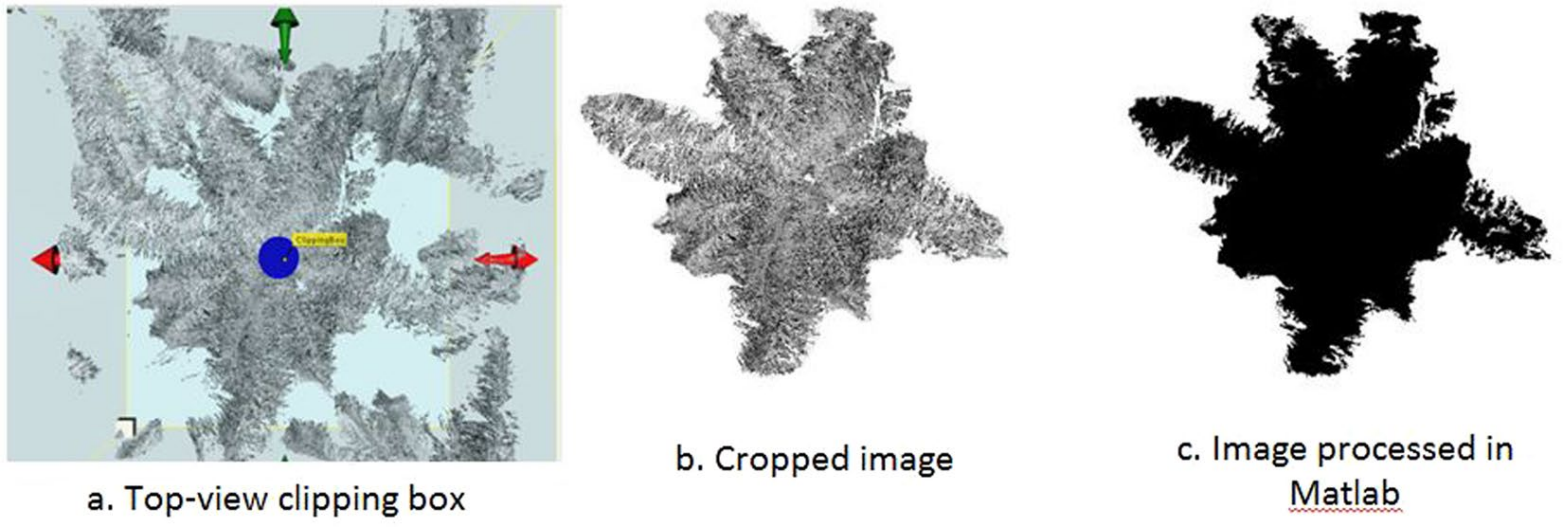
Steps of processing crown pixel (**a**–**c**).

The morphological operation (opening) was used to remove imperfections in the structure of the crown image. The opening operation smooths the outline of an object, clears the narrow bridges and also eliminates minor extensions present in the crown image by performing erosion followed by dilation. Dilation is a morphological operation that adds pixels to the boundary pixels. The dilation operation made the crown grow by size, while the erosion operation caused the crown to lose its size. Both operations use a small matrix structure based on the arrangement of ones and zeros in a pattern within the matrix known as a structuring element. A disk-shaped structuring element was used because the shape of the crown was arbitrary, smooth and not a polygon shape. After the area of crown was obtained, the number of pixels in the image was calculated. Figure 7 shows the process involved.

### Frond section

**Figure 8:**
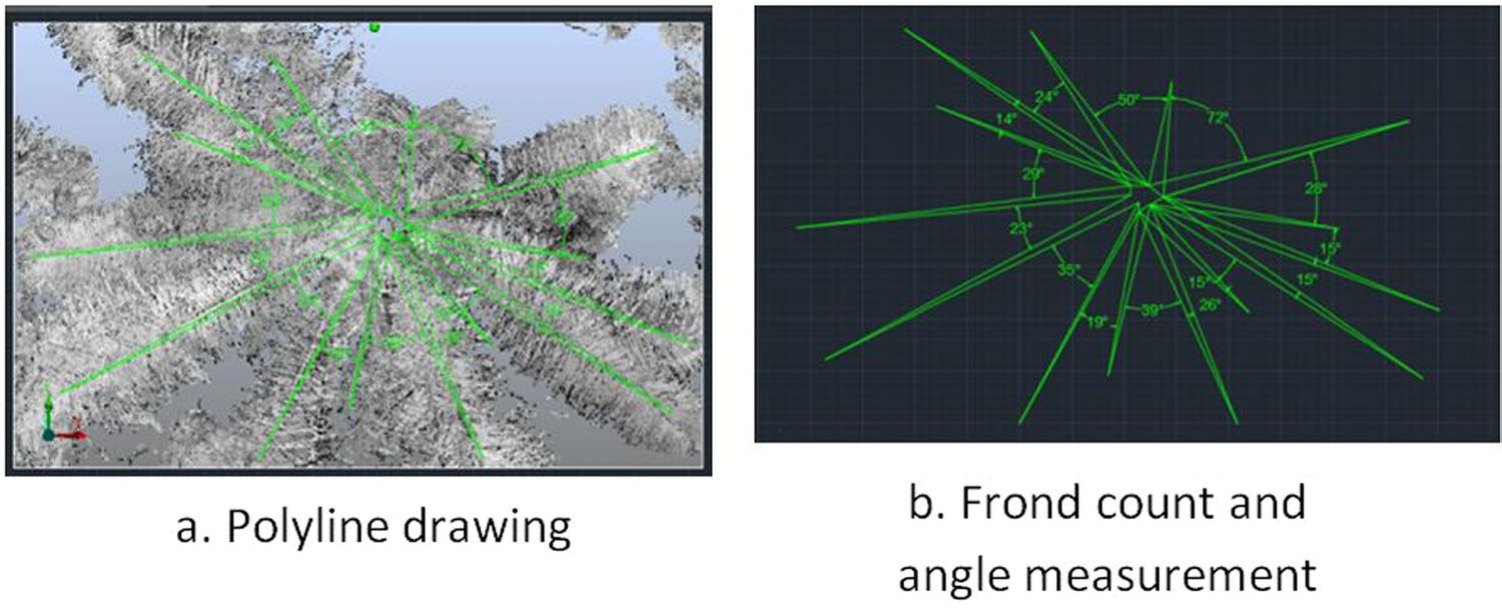
Steps of processing frond feature (**a**,**b**).

The method was similar to the crown pixel where a clipping box was created by placing a blue circle at the centre of the crown. The size of the clipping box was set differently according to the size of the frond. Then, the image of the frond from the top view was saved and exported to AutoCAD (Autodesk, Inc., California, USA). The ‘Draw’ and ‘Polyline’ tools were used to draw the line following the shape of the frond. After that, the ‘Dimension’ tool was selected to measure the angle between the fronds (in degrees). Lastly, the angle between the fronds and the number of fronds for every tree were recorded. Figure 8 illustrates the process of creating shapes of fronds in numbering orders.

### Canopy stratification

**Figure 9:**
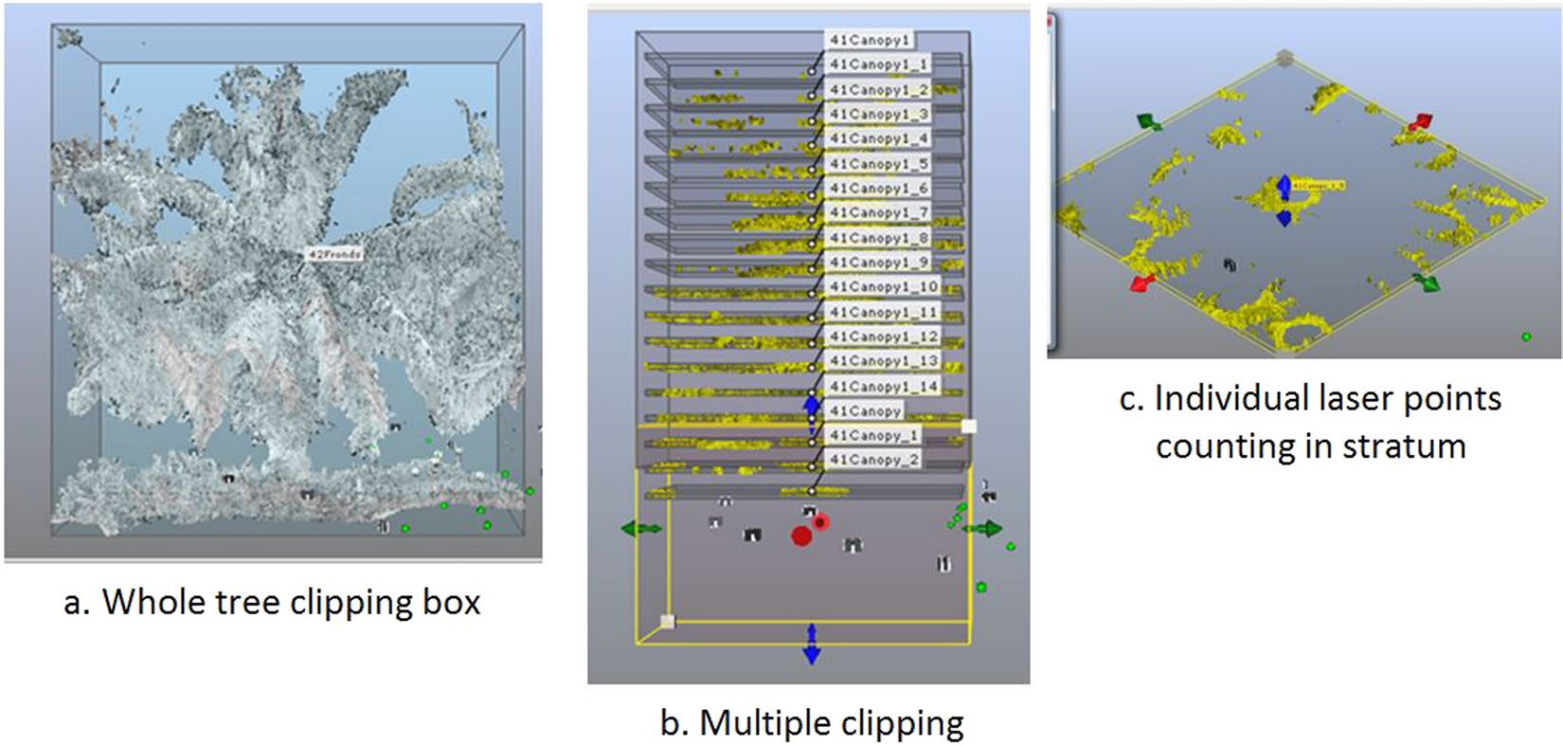
Steps of processing crown strata (**a**–**c**).

The initial point clouds from each tree consisted of a large number of laser points and created difficulties with data processing. Thus, to reduce the number of points in the cloud the data was segmented using the stratification method. The method was applied by implementing a series of horizontal planes with equal intervals and equal sized boxes (also known as voxels) along the vertical direction, resulting in many strata perpendicular to the vertical direction. A clipping box was created by placing a blue circle at the centre of the canopy or trunk, which looked hollow from the top-view. The dimensions, length and width of the clipping box were set to 6 m. The 6 m dimensions were set for the purpose of minimising the overlapping fronds from the trees located next to it. Then, the cursor of the clipping box was moved to the uppermost visible part (also known as top) of the tree canopy and moved down to the bottom limit where no fronds were visible in the clipping box. The ‘Create Clipping Boxes along an axis’ operation created multiple boxes along the vertical axis below the top part of the canopy section. The z-axis was chosen for the vertical direction and a negative sign was chosen for the downward direction of the clipping. The number of clipping boxes was set to 17. Based on the literature^48,49^ the thickness of the clipping box was set to 0.1 m and the space between the boxes was set to 0.5 m. The 0.5 m space between the boxes was selected arbitrarily but intended to minimise the point associated with the conversion from 3D real space into 2D model space. Then, the data (point value) was exported to a ‘PTS’ file and was opened using Notepad software (Microsoft, Washington, USA). The point value is the number of laser points that indicates the density of the canopy strata. Figure 9 shows the steps involved in crown stratification.

### Statistical Analysis and classification model

JMP software (SAS Institute, North Carolina, USA) was used to analyse the parameters using one-way analysis of variance (Kruskal-Wallis test) to check the significant different between the levels. Classification models were developed and used to detect the severity levels of BSR using all the biometrics extracted from TLS data. The regression coefficients using single and combinations of physical properties were calculated with the help of statistical software: Design Expert (version 10, Stat-Ease Inc., Minneapolis, USA), and the adequacy of the model was tested by using the analysis of variance (ANOVA). “User-Defined” design of experiments and “Numeric Factor” were chosen because they have the flexibility in modifying the model and all properties are in numeric values.

Data of this study were divided into three datasets: training, testing and validation. Twenty eight, twelve and forty oil palm trees taken from all healthiness levels were used for training, testing and validation datasets, respectively. Training datasets were used as an input to build a classification model acquired from Design Expert software. Each statistical model has a unique equation to represent the model. The equations were then transferred to Microsoft Excel (Microsoft, Washington, USA) and the models were generated using the testing datasets to obtain the mean and standard deviation for the models. The range for each class was considered by trial and error using the mean, standard deviation, maximum and minimum values. The classification models were then validated with different set of oil palm trees to determine the ability of the model to classify the oil palm trees, according to its healthiness level.

## Conclusion

This paper presented a new method used to classify four healthiness level conditions of oil palm trees due to *Ganoderma boninense* infection using physical properties of oil palm trees based on the differences in the canopy biometrics extracted from TLS scanner. Five parameters were used for the analysis: S200, S850, crown pixel, frond angle and frond number. One-way analysis of variance using Kruskal-Wallis test for all parameters showed all levels to be significantly different with p-value less than 0.05. Consequently, it can be concluded that combination parameters of linear model consisting of frond number, frond angle and canopy strata at 200 cm from the top was the best model compared to other combined parameter models. It gave an average accuracy of 80% for severity level classification and 86.67% accuracy for healthy-unhealthy classification. Furthermore, it performed better compared to the classification severity using single parameter, i.e. frond number. The method comprising of the classification model of canopy properties extracted from TLS data could be established for early detection of the BSR disease as early as T1 and could also be used to classify the level of BSR disease severity with high accuracy.

Based on this study, it can be concluded that the TLS remote sensing data offer accurate physical features of oil palm trees. The study has solved the research gap on the use of TLS in oil palm study for disease detection. The growing availability and quality of LiDAR data, combined with the method of characterising individual oil palm trees, offers potential for such data to play important roles in research and precision agriculture applications. Continuing technological advances are likely to result in the increased availability and resolution of TLS. In future, scanners with an advanced and higher capability that are able to scan a whole plantation area using a single scan with comparably lower cost, coupled with *in-situ* data processing facilities, could be developed. The system could be utilised to establish an accurate and rapid oil palm health map for real-time disease detection.

## Supplementary information

Fig. S1.
