# Supplementary material for: Application of Ground-Based LiDAR for Analysing Oil Palm Canopy Properties on the Occurrence of Basal Stem Rot (BSR) Disease
Source: Sci Rep. 2020 Apr 15;10:6464. doi: 10.1038/s41598-020-62275-6 (PMC7160211; doi:10.1038/s41598-020-62275-6)
Supplement: Supplementary file 1 — Fig. S1. [file 41598_2020_62275_MOESM1_ESM.docx]

**Application of Ground-Based LiDAR for Analysing Oil Palm Canopy Properties on the Occurrence of Basal Stem Rot (BSR) Disease**

Nur H. Azuan ^[[1]](#footnote-1)^, Siti Khairunniza-Bejo ^1,2^*, Ahmad F. Abdullah ^1,2^, Muhamad S. M. Kassim ^1,2^, Desa Ahmad ^1^, Aiman N.N. Azmi^1^

Department of Biological and Agricultural Engineering, Faculty of Engineering, Universiti Putra Malaysia, 43400 UPM Serdang, Selangor, Malaysia

^2^Smart Farming Technology Research Centre, Universiti Putra Malaysia, 43400 UPM Serdang, Selangor, Malaysia

*Correspondence: skbejo@upm.edu.my

The possible locations of laser scanner in the oil palm plantation for a large area implementation (Figure S1).

**Key**

** Oil palm tree**

**TLS scanner**

**Supplementary Fig. S1ǀ** The positions of laser scanner for large area implementation

1. [↑](#footnote-ref-1)
